# Correlation of circulating C1q and C1q-binding adiponectin concentrations with aging in males: a preliminary report

**DOI:** 10.1186/1758-5996-5-17

**Published:** 2013-03-27

**Authors:** Hideaki Nakatsuji, Ken Kishida, Hironori Kobayashi, Tohru Nakagawa, Tohru Funahashi, Iichiro Shimomura

**Affiliations:** 1 Department of Metabolic Medicine, Graduate School of Medicine, Osaka University, Osaka 565-0871, Japan; 2 Kishida Clinic, Osaka 560-0021, Japan; 3 Department of Research and Development, Diagnostic Division, Otsuka Pharmaceutical Co., Ltd, Tokushima 771-0195, Japan; 4 Hitachi, Ltd. Hitachi Health Care Center, Hitachi, Ibaraki, 317-0076, Japan; 5 Department of Metabolism and Atherosclerosis, Graduate School of Medicine, Osaka University, Osaka 565-0871, Japan

**Keywords:** Adiponectin, C1q, C1q-binding adiponectin, Aging

## Abstract

**Background:**

Atherosclerosis is an age-related disease. Adiponectin and C1q form a protein complex in human blood, and that serum C1q and C1q-binding adiponectin (C1q-APN) concentrations can be measured. We investigated circulating C1q and C1q-APN levels in Japanese men including elderly men.

**Findings:**

The study subjects were 509 Japanese men including elderly men. Serum levels of total adiponectin (Total-APN), high-molecular weight-adiponectin (HMW-APN), C1q-APN and C1q were measured by enzyme-linked immunosorbent assay. Total-APN, HMW-APN and C1q-APN, but not C1q, correlated significantly and positively with aging (r=0.26, r=0.24, r=0.17, p<0.01, respectively). The HMW-APN/Total-APN ratio correlated significantly and positively with aging (r=0.14, p<0.01). The C1q-APN/Total-APN ratio and C1q-APN/HMW-APN ratio correlated significantly and negatively with aging (r=−0.17, p<0.01, r=−0.12, p=0.01). C1q-APN/C1q correlated significantly and positively with aging (r=0.09, p=0.03). Multiple regression analysis identified age and body mass index as significant determinants of C1q-APN.

**Conclusions:**

The present study demonstrates that serum HMW-APN, C1q-APN, and Total-APN, but not C1q, correlated positively with aging. These preliminary results could form the basis for future research.

**Trial registration:**

Clinical Trial Registration Number: UMIN000004318

## Background

Atherosclerosis is an age-related disease [1]. Complement C1q activates canonical Wnt signaling and promotes aging-related phenotypes [2]. Wnt signaling also inhibits adipogenesis [3]. Adiponectin, which was identified by our group [4], has anti-atherosclerotic and anti-inflammatory properties in experimental animals and cells [5,6]. Adiponectin binds C1q *in vitro*, which induces activation of the classical complement pathway [7]. Our group reported recently that adiponectin and C1q form a protein complex in human blood, and that serum C1q and C1q-binding adiponectin (C1q-APN) concentrations can be measured using a new assay [8]. In the present study, we investigated circulating C1q and C1q-APN levels in Japanese men including elderly men.

## Subjects and methods

### Participants

The study subjects (Victor-J study: UMIN 000004318) were 509 Japanese men; 500 male employees, who underwent annual health checkup in 2010 at Hitachi Ltd (Ibaraki, Japan) [average age; 55±9 years (30–74), 30′s; n=41, 40′s; n=101, 50′s; n=229, 60′s; n=98, 70′s; n=31], and 9 residents of a geriatric health services facility at Minoh no Sato (Osaka, Japan) [average age; 84±11 years (71–100), 70′s; n=4, 80′s; n=2, 90′s; n=2, 100′s; n=1]. The present study was approved by the human ethics committees of Osaka University, Hitachi Health Care Center and Otsuka Pharmaceutical Co., Ltd.. Written informed consent was obtained from all subjects.

### Anthropometric data and laboratory tests

Anthropometric variables [height, weight] were measured in the standing position and and body mass index (BMI) was calculated [=weight (kg) / height (m)^2^]. Systolic and diastolic blood pressures (SBP, DBP) were measured with a standard mercury sphygmomanometer on the left or right arm in the standing position after at least 5-minute rest. Venous blood samples were collected in the morning after overnight fast for measurements of serum creatinine, total cholesterol (T-cho), triglyceride (TG), high-density lipoprotein-cholesterol (HDL-C), glucose, and HbA1c (Japan Diabetes Society [JDS]). The value of HbA1c (%) was estimated as the National Glycohemoglobin Standardization Program (NGSP) equivalent value (%), calculated by the formula HbA1c (%) = HbA1c (JDS, %) + 0.4%. Low-density lipoprotein-cholesterol (LDL-C) was calculated using the Friedewald formula. Estimated GFR (eGFR) was calculated using the simplified Modification of Diet in Renal Disease equation modified by the appropriate coefficient for Japanese populations by gender, as described previously [9]. Serum samples were obtained at baseline from each participant and stored promptly at −20°C. After thawing the samples, serum levels of total adiponectin (Total-APN) and high-molecular weight-adiponectin (HMW-APN) were measured by enzyme-linked immunosorbent assay (ELISA) (Human adiponectin ELISA kit, Human HMW-adiponectin ELISA kit, Otsuka Pharmaceutical Co. Tokushima, Japan) [4,10]. Serum levels of C1q-APN and C1q were measured by our manufactured ELISA, as reported previously by our group [8]. The intra- and inter-coefficients of variation (CV) for C1q-APN ELISA are below 4.6% and 6.7%, respectively. The intra- and inter-CV for C1q ELISA are below 4.6% and 5.0%, respectively. Smoking status was collected through a self-questionnaireis (non-, ex- or current-) and Brinkman index (daily number of cigarettes × years). Medical history was surveyed by using a self-administered questionnaire including diabetes, dyslpidemia, hypertension, and past history of myocardial infarction, angina, stroke [11]. Heart failure was defined as plasma brain natriuretic peptide levels >100 pg/mL, and renal failure was defined as plasma eGFR <60 mL/min.

### Statistical analysis

Data are expressed as mean±SD. Relationships between two continuous variables were analyzed using scatter plots and Pearson’s correlation coefficient. The correlations between C1q-APN, C1q and clinical features, were first analyzed by univariate regression analysis and then by multivariate regression analysis. In all cases, *p* values <0.05 were considered statistically significant. All statistical analyses were performed with The Statistical Package for Social Sciences (version 11.0, SPSS Inc, Chicago, IL).

## Results

**Figure 1:**
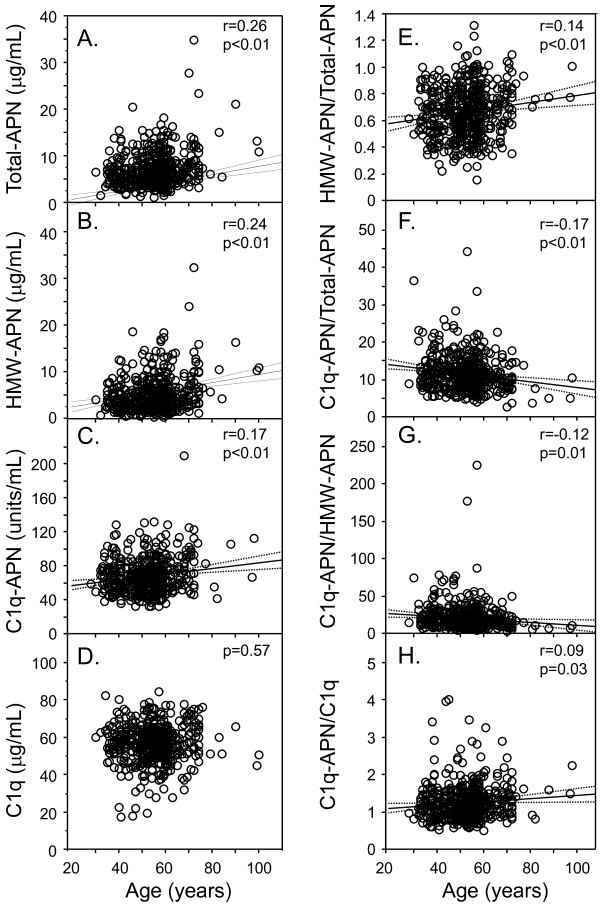
**Correlations of circulating levels of Total-APN, HMW-APN, C1q-APN, C1q, HMW-APN/Total-APN, C1q-APN/Total-APN, C1q-APN/HMW-APN, and C1q-APN/C1q with age.** Abbreviations as in Table 1. Relationships between two continuous variables were analyzed by Pearson’s correlation analysis. Solid lines: regression lines, Dotted lines: 95% confidence interval (CI) of predicted mean together with reference lines indicating the baseline for each adiponectin parameter and C1q levels (0% change).

Table 1 shows the characteristics of all male subjects. Total-APN, HMW-APN and C1q-APN, but not C1q, correlated significantly and positively with aging (Figure 1A-D). The HMW-APN/Total-APN ratio correlated significantly and positively with aging (Figure 1E). The C1q-APN/Total-APN ratio and C1q-APN/HMW-APN ratio correlated significantly and negatively with aging (Figure 1F, G). C1q-APN/C1q correlated significantly and positively with aging (Figure 1H).

**Table 1:** Baseline characteristics

**n (males)**	**509**
Age, years	54±10 (30–100)
Body mass index (BMI), kg/m^2^	24.1±3.0 (14.8−36.5)
Hypertension, % (medication, %)	42.2 (23.8)
Systolic blood pressure (SBP), mmHg	123±12 (91–174)
Diastolic blood pressure (DBP), mmHg	78±8 (47–100)
Diabetes, % (medication, %)	33.6 (4.9)
Fasting blood glucose (FBG), mg/dL	109±18 (64–284)
Hemoglobin A1c (HbA1c), (NGSP), %	5.8±0.6 (4.5−10.2)
Estimated glomerular filtration rate (eGFR), mL/min	69.4±12.8 (28.6−130.5)
Dyslipidemia, % (medication, %)	40.9 (12.4)
Total cholesterol (T-cho), mg/dL	204±33 (104–332)
Triglyceride (TG), mg/dL	140±94 (35–871)
High-density lipoprotein-cholesterol (HDL-C), mg/dL	56±13 (27–97)
History of myocardial infarction, angina, stroke, heart failure, and renal failure, %	0.8/0.2/0.6/0.6/23.8
Smoking (none-/ex--/current-), %	26.2/48.0/25.8
Brinkman index	383±352 (0–2000)
Serum Total-APN, μg/mL	6.8±3.6 (1.1−34.8)
Serum HMW-APN, μg/mL	4.9±3.6 (0.2−32.3)
Serum C1q-APN, units/mL	67.8±19.8 (31.6−209.3)
Serum C1q, μg/mL	56.7±9.8 (17.3−84.2)
Serum HWM-APN/Total-APN ratio	0.7±0.2 (0.2−1.3)
Serum C1q-APN/Total-APN ratio	11.5±4.6 (2.6−44.3)
Serum C1q-APN/HMW-APN ratio	20.4±17.0 (2.8−225.3)
Serum C1q-APN/C1q ratio	1.2±0.5 (0.5−4.0)

Data are mean±SD (range), or number of subjects.

APN; adiponectin, HMW; high-molecular weight.

Next, univariate regression analysis was used to evaluate the relationship between C1q-APN, C1q and clinical features (Table 2). C1q-APN correlated positively with age and HDL-C, and negatively with BMI, and log TG. Multiple regression analysis of age, BMI, log-TG, HDL-C and C1q-APN identified age and BMI as significant determinants of C1q-APN. On the other hand, C1q correlated positively with BMI, SBP, DBP, T-cho, log TG and negatively with HDL-C. Multiple regression analysis of BMI, SBP, DBP, T-cho, log-TG and HDL-C identified SBP and HDL-C as significant determinants of C1q.

**Table 2:** Correlations between C1q-APN, C1q and clinical features in men

	**Log C1q-APN**			**C1q**		
	***Univariate***		***Multivariate***	***Univariate***		***Multivariate***
	**r**	**p**	**p**	**r**	**p**	**p**
Age	**0.1612**	**0.0002**	**0.0076**		0.5661	
BMI	**−0.2490**	**<0.0001**	**<0.0001**	**0.1549**	**0.0004**	0.2761
SBP		0.7847		**0.2074**	**<0.0001**	**0.0098**
DBP		0.7006		**0.1975**	**<0.0001**	0.2140
FBG		0.6776			0.0678	
HbA1c		0.1992			0.7861	
eGFR		0.6774			0.4865	
T-cho		0.1970		**0.1095**	**0.0149**	0.0910
Log-TG	**−01483**	**0.0009**	0.1219	**0.1949**	**<0.0001**	0.2585
HDL-C	**0.1095**	**0.0122**	0.7865	**−0.1845**	**<0.0001**	**0.0043**
Brinkman index		0.2472			0.9811	
adjusted r^2^			0.071			0.089

Data of C1q-APN and TG levels showed skewed distribution and were log-transformed before analysis. Significant level was set at p<0.05 (bold typeface). Multiple regression analysis was conducted to identify those parameters that significantly contributed to C1q-APN or C1q. Parameters with *p*<0.05 were subsequently entered into the regression analysis as independent variables (bold typeface). Abbreviations as in Table 1.

## Discussion

This is the first report showing that serum HMW-APN and C1q-APN as well as Total-APN, but not C1q, correlated with aging, and that multiple regression analysis identified age as well as BMI as significant determinants of C1q-APN.

As one possible mechanism, three major factors are involved in the control of circulating Total-APN levels; gender, body weight and renal function [4,12,13]. The present study found that advanced age is associated with decreased body weight (r=−0.1612, p=0.0003) and reduced renal function (r=−0.2168, p<0.0001, data not shown). These findings may provide an explanation for the increased adiponectin levels in elderly men.

Another possible mechanism is dysregulated production of adipocytokines in elderly subjects, especially hyperadiponectinemia, which is associated with normal aging process [14]. The data suggests that age-related lipoatrophy may be the major factor for adipose tissue dysfunction in advanced age. Transgenic expression of human adiponectin blocked excessive fat accumulation and reduced morbidity and mortality rates in mice fed high-calorie diet, and prolonged the lifespan of normal mice, by inhibiting AKT signaling and chronic inflammation [15]. Based on our recent studies [8,16,17], low Total-APN and high C1q-APN/Total-APN correlated with coronary artery disease, suggesting that adiponectin may protect against the development of atherosclerosis through the activation of the complement system and that it can modulate the self-defense system through its binding with C1q in the peripheral circulation. The present study demonstrates that high Total-APN and low C1q-APN/Total-APN ratio correlated positively, but not strongly, with aging (Figure 1). Recent study suggests that centenarians possess a different adiponectin isoform pattern [18]. Taken together, high Total-APN levels and low C1q-APN/Total-APN ratio seems to inhibit aging-related phenotypes including atherosclerosis, and, at least partly, may live longer than others. Further longitudinal studies of larger populations of elderly subjects are required.

## Conclusion

The present study demonstrated that serum HMW-APN, C1q-APN, and Total-APN, but not C1q, correlated positively with aging.

### Study limitations

Certain limitations of this study must be considered. First, this is a cross-sectional study, making it difficult to establish a cause-effect relationship. Further prospective studies should be conducted to analyze this relationship. Second, all patients in this study were Japanese men and any differences from other ethnic groups are unknown. The results may not be applicable to females or non-Japanese populations. The other preliminary study analyzed the correlation of circulating C1q-APN concentrations with aging in Japanese females (n=144, average age (range), 45±10 years (23–64), 20′s; n=10, 30′s; n=36, 40′s; n=46, 50′s; n=46, 60′s; n=6). Total-APN, but not C1q-APN, correlated positively with age (r=0.20, p=0.013). However, Adamczak et al. reported that plasma adiponectin concentration in females did not change significantly with age [19]. In our preliminary study, the number of over 60′s elderly subjects may be still small, and further investigations are required. Third, the current study did not include the effects of physical activity, alcohol intake, socio-economic status, marital status, dietary habit and use of pharmacotherapy. Fourth, medical history surveyed by using a self-administered questionnaire remains some ambiguities. Finally, in Japan, employees shall be liable to undergo annual health checkup until the age of retirement (65 years). On the other hand, the elderly aged 65 or over have no obligation to undergo annual health checkup in medical insurance system. In Japan, apparently healthy elders were residents of a geriatric health services facility who have few serious diseases, but not hospitalized patients. We decided residents of a geriatric health services facility to participate in this study. The number of over 70′s elderly subjects may be still small. Therefore the present study has a selection bias. Further investigations might be required to confirm the present results.

## Abbreviations

C1q-APN: C1q-binding adiponectin; CV: Coefficients of variation; ELISA: Enzyme-linked immunosorbent assay; HDL-C: High-density lipoprotein-cholesterol; HMW-APN: High-molecular weight-adiponectin; T-cho: Total cholesterol; TG: Triglyceride; Total-APN: Total adiponectin.

## Competing interests

Tohru Funahashi is a member of the “Department of Metabolism and Atherosclerosis”, a sponsored course endowed by Kowa Co. Ltd. The company has a scientific officer who oversees the program. All other authors declare no competing interests. Human serum C1q-binding adiponectin complex assay is under patent application in Japan.

## Authors’ contributions

HN and KK researched and analyzed data. KK also participated in the concept and design of the study, interpretation of data and reviewed/edited the manuscript. HK analyzed the data. TN recruited the patients and collected the data. TF and IS contributed to the discussion and wrote the manuscript. All authors read and approved the final version of the manuscript.
